# Olmesartan Associated Sprue-Like Enteropathy and Colon Perforation

**DOI:** 10.1155/2014/494098

**Published:** 2014-03-04

**Authors:** Mahmoud Abdelghany, Luis Gonzalez, John Slater, Christopher Begley

**Affiliations:** ^1^Department of Medicine, Conemaugh Memorial Medical Center, 1086 Franklin Street, E3 building, Johnstown, PA, 15905, USA; ^2^Department of Pharmacy, Conemaugh Memorial Medical Center, 1086 Franklin Street, E3 building, Johnstown, PA, 15905, USA

## Abstract

We are reporting a unique case of olmesartan associated severe sprue-like enteropathy in a 52-year-old woman who presented to our hospital complaining of severe abdominal pain and nausea. At the emergency department she suffered from a cardiac arrest and was found to have a colon perforation. The patient was treated conservatively without surgical intervention and olmesartan was discontinued. After one month, she had complete resolution of her symptoms.

## 1. Introduction

Olmesartan is an angiotensin receptor blocker (ARB) approved for the treatment of hypertension in 2002. FDA reported olmesartan associated sprue-like enteropathy via a MedWatch alert in July 2013 after a case series of 22 patients was reported by Mayo Clinic [1]. Since that time only very few literatures discussed this adverse effect [2–4]. To our knowledge, this is the first reported case of olmesartan associated sprue-like enteropathy to present with colon perforation possibly secondary to the severity of the enteropathy.

## 2. Case Description

A 52-year-old woman presented to our hospital complaining of severe abdominal pain and nausea. She reported recurrent mild abdominal pain, bloating, nausea, occasional vomiting, and severe nonbloody diarrhea with 20 evacuations a day for one year. She had a 45 pound weight loss within six months. She denied any other symptoms suggestive of local or systemic infections, recent travels or sick contacts, changes in her diet habit, or medications within the last year.

**Figure 1:**
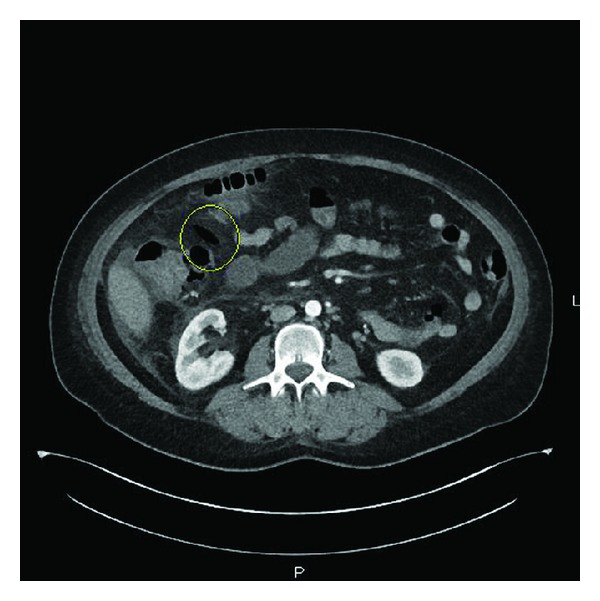
Contrast-enhanced CT abdomen showing wall thickening in the ascending colon with pericolonic stranding pattern and adjacent free air (circle) suggestive of colon perforation.

**Figure 2:**
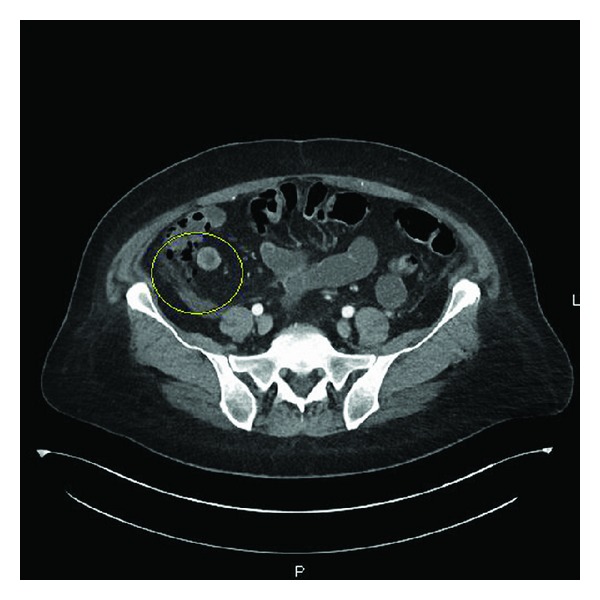
Contrast-enhanced CT abdomen showing wall thickening in the ascending colon with pericolonic stranding pattern and adjacent tiny foci of free air (circle) suggestive of colon perforation.

Vital signs were within normal range. Physical exam revealed generalized abdominal tenderness with guarding and signs of dehydration. At the emergency department, the patient suffered from a cardiac arrest. She was successfully resuscitated and admitted to the intensive care unit. Laboratory investigations showed potassium 2.9 mMol/L, bicarbonate 17 mMol/L, blood urea nitrogen 7 mg/dL, creatinine 0.9 mg/dL, white blood count 12.1 × 10³/*μ*L, hemoglobin 13.8 gm/dL, and albumin 2.4 gm/dL. CT scan of the abdomen showed ascending colon inflammation and evidence suggestive of perforation (Figures 1 and 2). She was treated conservatively without surgical intervention. A repeat abdominal CT scan revealed improving of the colonic inflammation and sealing of the perforation.

An extensive investigation for chronic diarrhea over the past year was performed, including serum thyroid stimulating hormone and stool cultures, *Clostridium difficile* toxin assay, ova, parasites, osmolality, and electrolytes that were unremarkable. Celiac disease was excluded by negative conventional serology tests (tissue transglutaminase antibodies, endomysial antibodies and antienterocyte antibodies) and the absence of a clinical response to a gluten-free diet. Management including an opioid-receptor agonist, corticosteroids, azathioprine, and antibiotic therapy failed to relieve her symptoms. Endoscopic biopsies from the stomach and colon showed inflammatory changes and lymphoid aggregation with no subepithelial collagen depositions. *Helicobacter pylori* testing of the specimens was negative.

She had been on olmesartan (20 mg/day) for hypertension for 3 years. Olmesartan associated enteropathy was suspected and the drug was discontinued and replaced by lisinopril. One month later, she had complete resolution of the abdominal discomfort and diarrhea. After 5 months, the patient continued to be asymptomatic with no gastrointestinal manifestations.

## 3. Discussion

Although olmesartan has been approved for the treatment of hypertension since 2002, sprue-like enteropathy, as an adverse effect, was not known until 2012 when Rubio-Tapia et al. [1] reported 22 patients who suffered from this adverse effect. The patients usually present with chronic diarrhea, vomiting, abdominal pain, bloating, and a median of a 40 pound weight loss. Dehydration and acute renal failure have also been reported [1]. All cases showed marked clinical improvement after discontinuing olmesartan.

Olmesartan associated enteropathy may be differentiated from celiac disease by the absence of tissue transglutaminase and endomysial and antienterocyte antibodies with absence of response to a gluten-free diet. Histological findings include intestinal villous atrophy with mucosal inflammation and lymphoid aggregation [1]. Rubio-Tapia et al. [1] reported pathologic evidence of involvement of the stomach and colon, with lymphocytic aggregation, suggesting that this disorder may affect the entire gastrointestinal tract. The mechanisms underlying olmesartan associated enteropathy are unknown. One proposed mechanism is related to a cell-mediated immune response that damages the small intestinal brush border. ARBs have been suggested to have inhibitory effects on transforming growth factor *β*, which is responsible for gut immune homeostasis and maintaining a normal balance between proinflammatory and anti-inflammatory factors [5, 6]. HLA DQ2 and DQ8 testing were not performed in our patient due to her excellent clinical response after discontinuing olmesartan. Although HLA D Q2/Q8 have good negative predictive value for celiac disease [7], they have poor positive predictive value for celiac disease and olmesartan associated enteropathy [1, 7]. Deliberate rechallenge test with olmesartan was not indicated because of the life-threatening nature of the disease. Complete resolution of her symptoms with absence of clinicopathological evidences of other diseases associated with enteropathy suggests that the association is not likely to be a chance.

Recognition of olmesartan associated enteropathy explored a diagnosis of a good proportion of unclassified sprue (US), also known as unspecified enteropathy. In 2012, Pallav et al. [8] reported a diagnosis of US in 33% of the patients with seronegative villous atrophy. In 2013, and after the recognition of olmesartan associated enteropathy, DeGaetani et al. [3] reported a percentage of 14% for the same disease that would increase to 30% if olmesartan associated enteropathy was still unknown.

To our knowledge, our patient is the first to present with colon perforation possibly due to the severity of the enteropathy. Since this condition is serious and likely to be underreported, different approaches are warranted to alert the clinical community of this problem. Until it is determined whether other ARBs can result in a similar adverse reaction, clinicians should remain vigilant for development of sprue-like enteropathy in patients taking ARBs.
